# Adrian Grant’s pioneering use of evidence synthesis in perinatal medicine, 1980–1992

**DOI:** 10.1186/s12978-018-0518-3

**Published:** 2018-05-15

**Authors:** Iain Chalmers

**Affiliations:** James Lind Initiative, Oxford, UK

**Keywords:** Systematic reviews, Randomized controlled trials, Perinatal medicine, Evidence based practices

## Abstract

Systematic reviews of existing research are needed to help reduce the enormous amount of wasted resources in biomedical research. Whether already available or needed but unavailable, systematic reviews are a key element in prioritising questions for new research, and for informing the design of additional studies. One of the most important of Adrian Grant’s many contributions was to recognise this a decade before it began to become more widely accepted. In this sphere, as well as in many others, he was a real pioneer.

## The National Perinatal Epidemiology Unit (NPEU)

Adrian Grant died in 2015 at the age of 67 from ocular melanoma. He had several distinguished careers, but the one with which I am most intimately familiar concerns his time as the epidemiologist at the National Perinatal Epidemiology Unit (NPEU), in Oxford, between 1980 and 1994.

The NPEU had got off to a rocky start in 1978. It was created by the UK government in response to expressions of public concern that national trends in perinatal mortality - and by inference, long term morbidity among survivors - compared unfavourably with experience in some other countries. The creation of the Unit was announced in a ministerial answer to a parliamentary question, in which it was stated that “The broad remit of the unit is to conduct epidemiological research in the perinatal field with a view to providing information which can promote effective use of resources in the perinatal health services” (Hansard 5 July 1978).

The proposed programme of work of the Unit had six elements. One of these was to consider plans for a 4th National Perinatal Mortality Survey, possibly to be launched in 1982. An experienced perinatal epidemiologist, Jean Golding (née Fedrick), was recruited to the Unit to look into this possibility. An account of what happened subsequently has been published in a book by Helen Pearson [1]. In brief, Jean Golding’s ambitious plans did not find favour with the Unit’s advisors. Undaunted, Jean moved to Bristol and established there what has been a very successful birth cohort – the Avon Longitudinal Study of Parents and Children [2].

The Unit’s post of epidemiologist having become vacant, it was clearly important to recruit someone likely to be able to take on one or more of the five remaining elements in its proposed programme of work. One of these was to develop a programme of randomized trials, a field of perinatal research in which there was no substantive focus of expertise anywhere in the world. After all, Archie Cochrane had then recently awarded obstetrics and gynaecology ‘the wooden spoon’ for being the medical speciality least informed by scientific evidence [3]. Who might be willing to try to create a focus of expertise in randomised trials at the NPEU?

Fortunately for the NPEU and for the users of perinatal health services, Adrian Grant – who had qualifications in both obstetrics and epidemiology - accepted the invitation to join the Unit to take up this challenge. Adrian made several pioneering contributions to the NPEU’s work during his time there. The theme on which I wish to focus, however, concerns the use of systematic reviews of controlled trials to inform the development and portfolio of the Perinatal Trials Service that he created with Diana Elbourne and others.

## Evidence-based research at the NPEU

Embarking on research without reviewing systematically evidence of what is already known - particularly when the research involves people or animals - is unethical, unscientific, and wasteful [4]; yet this practice is widespread [5]. The antidote to this form of academic misconduct has recently been named Evidence-Based Research [6]. At last the principle of evidence-based research is beginning to be taken more seriously, not only by academic lobbyists (www.ebrnetwork.org), but also by research funders and research ethics committees. The National Institute for Health Research in England, for example, advises applicants for support of new primary research as follows:

“Where a systematic review already exists that summarizes the available evidence this should be referenced, as well as including reference to any relevant literature published subsequent to that systematic review. Where no such systematic review exists it is expected that the applicants will undertake an appropriate review of the currently available and relevant evidence (using as appropriate a predetermined and described methodology that systematically identifies, critically appraises and then synthesizes the available evidence) and then present a summary of the findings of this in their proposal. All applicants must also include reference to relevant ongoing studies, e.g. from trial registries.” [7]

Among research regulators, the guidance for researchers issued by the Health Research Authority in the UK now states “Any project should build on a review of current knowledge. Replication to check the validity of previous research is justified, but unnecessary duplication is unethical” [8].

The principle of Evidence-Based Research – albeit without using this term – was incorporated in the work of the NPEU from the outset. This was given impetus by Archie Cochrane’s 1979 criticism of the medical profession for not having organised “a critical summary, by speciality or subspeciality, adapted periodically, of all relevant randomized controlled trials” [3]. How did the NPEU and its friends in the UK and abroad respond to this challenge?

A start had been made the previous year. At the Congress of the European Society of Perinatal Medicine in Vienna in 1978, I presented a systematic review and meta-analysis of data from three published and one unpublished randomised comparisons of different methods of intrapartum fetal monitoring [9]. These randomized trials had been identified through a formal search for such studies which I had started a few years earlier. Soon after Adrian’s arrival in the NPEU, he and I wrote to *The Lancet* drawing attention to the rationale for developing this register of randomised trials in perinatal medicine [10]. We wrote that:

“The register has already provided an invaluable data base for reviews of the safety and efficacy of interventions in perinatal medicine. It has enabled us to identify those areas which have been investigated with clinical experiments and, through these studies, it has provided an efficient introduction to the observational data. The section of the register concerned with as yet unpublished trials has been of particular interest to those considering or planning further randomised studies”.

We concluded our letter with a request:

“We appeal to those of your readers who have been or are currently involved in perinatal clinical trials either to send us copies of their key publications or to notify us of the existences of unpublished or as yet unfinished trials. We also urge authors (and journal editors) to include a reference to research methods in the summaries, which are now an element of most research reports. Lastly, we hope that staff at the National Library of Medicine in Washington will assist by more frequent use of the indexing descriptor ‘random allocation’ (which was introduced in 1978).”

The Perinatal Trials Register was subsequently published in book form by Oxford University Press, with support from the World Health Organisation [11], and it was made available the following year in electronic form [12].

The register proved valuable in the early 1980s during the preparation of a collection of reviews of interventions used during antenatal care [13]. Adrian contributed a chapter to this book on the effects of physical interventions intended to prolong pregnancy and increase fetal growth [14]. However, it was during this time that the 1978 systematic review and meta-analysis of intrapartum fetal monitoring trials [9] came to play an important role in Adrian’s life and the work of the NPEU more generally.

Among the total of just over two thousand babies born in the intrapartum fetal monitoring trials analysed in that review, thirteen had experienced seizures during the neonatal period. The distribution of these babies among the comparison groups was quite unlikely to have reflected chance (meta-analysis yielded a *P* value of less than .01): babies monitored using a combination of continuous fetal heart rate monitoring and acid base estimates if indicated were less likely to have experienced convulsions after delivery than were babies who had been monitored using alternative approaches [9]. This observation prompted Adrian to design, run and analyse a randomised trial involving over 10,000 women and babies to assess whether the result of the earlier systematic review and meta-analysis would be borne out. It was [15], and Adrian’s research led to the award of a Doctorate in Medicine by the University of Oxford.

This encouraging confirmation of a hypothesis which had arisen from an early meta-analysis led us to venture further with this approach. In a letter to *The Lancet* that I co-authored with Adrian and Diana we commented on the implications of the results of four controlled trials of the use of phenobarbitone used to try to prevent periventricular haemorrhage in prematurely born neonates [16]. We began our letter by presenting a summary relative risk (0.36) of the effect of the drug on haemorrhage, which suggested that it might reduce the risk of this very serious form of morbidity, but we observed that the statistic could easily reflect the play of chance. We concluded the letter with a warning:

“We hope that this analysis may serve as a reminder of the dangers of false inference from both non-randomised comparisons and small randomised trials. Many trials in the perinatal period require sample sizes larger than any single unit can generate within a reasonable length of time. A recognition of this reality…has resulted in more than a dozen neonatal units joining together to enter cases into the first truly collaborative trial of neonatal practice ever to have been mounted in Britain. The objective of this is to assess alternative ways of managing post-haemorrhagic ventricular dilatation – but there is no reason why it could not provide a framework for collaboration to address other clinically important questions.”

These principles became the basis for the Perinatal Trials Service established at the NPEU by Adrian and Diana.

## *Effective care in pregnancy and childbirth, the Oxford database of perinatal trials*, and the Cochrane Collaboration

The information contained in the evolving Perinatal Trials Register was used in preparing *Effectiveness and Satisfaction in Antenatal Care*, a book edited by Murray Enkin and me, to which Adrian contributed [14]. Between 1985 and 1989, the Register proved essential when Murray Enkin, Marc Keirse and I, with support from Adrian and other colleagues at the NPEU, established an international collaboration to prepare systematic reviews (and meta-analyses when appropriate) of controlled trials in pregnancy and childbirth [17]. Ninety seven contributors, from Australia, Belgium, Canada, Finland, France, Italy, Netherlands, Norway, South Africa, United Kingdom, United States of America, and Zimbabwe, collaborated to produce a massive 2-tome book entitled *Effective Care in Pregnancy and Childbirth* [18]. Adrian was the author or a co-author of eight chapters in the book - on evaluation of screening, ultrasound, fetal movement counting, cervical cerclage, management of preterm labour, monitoring the fetus during labour, repair of perineal trauma, and relief of perineal pain.

Because most books are out of date by the time they are published, we began publishing updated (and sometimes corrected) analyses in an electronic publication entitled *The Oxford Database of Perinatal Trials (ODPT).* This was distributed by Oxford University Press in 6-monthly issues of 5¼ inch floppy disks, the content of which had to be downloaded to the hard disk of a personal computer. As the storage capacity of floppy disks grew, *ODPT* could be distributed on two 3 half inch. floppy disks. Adrian was one of the people who contributed to this pioneering example of electronic publication.

These methods and the prototypes developed at the NPEU were influential in helping people to conceptualise an international collaboration to extend the methods used in creating *The Oxford Database of Perinatal Trials* to cover all of health care. Among those who recognised this potential was Michael Peckham, Director of the recently established NHS Research and Development Programme. In 1992, he agreed to fund (for an initial three years) a ‘Cochrane Centre’, “to collaborate with others, in the UK and elsewhere, to facilitate systematic, up-to-date reviews of randomised controlled trials of health care”. The Cochrane Centre was established in Oxford in October the following year, and convened the meeting in October 1993 at which the international Cochrane Collaboration was established [19].

It was at this time that Adrian decided that he was ready for a move, and he was appointed in 1994 to direct the Health Services Research Unit at Aberdeen University. Adrian’s pioneering use of evidence synthesis in perinatal medicine was reflected in his decision to convene and lead an international Cochrane Incontinence Group, with its editorial base in Aberdeen. The respect with which Adrian is held derives from his work in many different spheres. In the field of research synthesis it was reflected in his election to co-chair the Cochrane Collaboration between 2007 and 2009.

## Conclusion

I am not confident that the National Perinatal Epidemiology Unit would have survived had Adrian not joined it in 1980. I owe him more than I can express. I have illustrated the key role he played in establishing the NPEU’s contribution to the field of research synthesis, how his contributions to this field were internationally recognised, and that they remained with him until he died.

Anyone who does systematic reviews of research cannot fail to realise that, left to themselves, researchers do research which is of little or no relevance to the interests of the users of research [20]. Adrian acknowledged this problem in fostering the creation of the James Lind Alliance’s Research Priority Setting Partnership in urinary incontinence. This brought patients, carers and clinicians together to reach agreement on their shared priorities for research [21].

Systematic reviews of existing research are needed to help reduce the enormous amount of wasted resources in biomedical research (www.rewardalliance.net). Whether already available or needed but unavailable, systematic reviews are a key element in prioritising questions for new research, and for informing the design of additional studies. One of the most important of Adrian’s many contributions was to recognise this a decade before it began to become more widely accepted. In this sphere, as well as in many others, he was a real pioneer.
